# Is Metabolic Syndrome Predictive of Prevalence, Extent, and Risk of Coronary Artery Disease beyond Its Components? Results from the Multinational Coronary CT Angiography Evaluation for Clinical Outcome: An International Multicenter Registry (CONFIRM)

**DOI:** 10.1371/journal.pone.0118998

**Published:** 2015-03-03

**Authors:** Amir Ahmadi, Jonathon Leipsic, Gudrun Feuchtner, Heidi Gransar, Dan Kalra, Ran Heo, Stephan Achenbach, Daniele Andreini, Mouaz Al-Mallah, Daniel S. Berman, Matthew Budoff, Filippo Cademartiri, Tracy Q. Callister, Hyuk-Jae Chang, Kavitha Chinnaiyan, Benjamin Chow, Ricardo C. Cury, Augustin Delago, Millie J. Gomez, Martin Hadamitzky, Joerg Hausleiter, Niree Hindoyan, Philipp A. Kaufmann, Yong-Jin Kim, Fay Lin, Erica Maffei, Gianluca Pontone, Gilbert L. Raff, Leslee J. Shaw, Todd C. Villines, Allison Dunning, James K. Min

**Affiliations:** 1 Department of Medicine, University of British Columbia, Vancouver, BC, Canada; 2 Department of Radiology, University of British Columbia, Vancouver, BC, Canada; 3 Department of Radiology, Medical University of Innsbruck, Innsbruck, Austria; 4 Department of Imaging, Cedars-Sinai Medical Center, Los Angeles, California, United States of America; 5 Department of Radiology, NewYork-Presbyterian Hospital and the Weill Cornell Medical College, New York, New York, United States of America; 6 Department of Medicine, University of Erlangen, Erlangen, Germany; 7 Centro Cardiologico Monzino, IRCCS, Milan, Italy; 8 King Abdulaziz Cardiac Center, King Abdulaziz Medical City, Riyadh, Saudi Arabia; 9 Department of Medicine, Harbor UCLA Medical Center, Los Angeles, California, United States of America; 10 Department of Radiology, Giovanni XXIII Hospital, Monastier, Treviso, Italy; 11 Tennessee Heart and Vascular Institute, Hendersonville, Tennessee, United States of America; 12 Division of Cardiology, Severance Cardiovascular Hospital and Severance Biomedical Science Institute, Yonsei University College of Medicine, Yonsei University Health System, Seoul, South Korea; 13 William Beaumont Hospital, Royal Oaks, Michigan, United States of America; 14 Department of Medicine, University of Ottawa, Ottawa, ON, Canada; 15 Baptist Cardiac and Vascular Institute, Miami, Florida, United States of America; 16 Capitol Cardiology Associates, Albany, New York, United States of America; 17 Division of Cardiology, Deutsches Herzzentrum Munchen, Munich, Germany; 18 Medizinische Klinik I der Ludwig-Maximilians-Universität München, Munich, Germany; 19 University Hospital, Zurich, Switzerland; 20 Seoul National University Hospital, Seoul, South Korea; 21 Department of Radiology, Erasmus Medical Center, Rotterdam, The Netherlands; 22 Department of Medicine, Emory University School of Medicine, Atlanta, Georgia, United States of America; 23 Department of Medicine, Walter Reed National Military Medical Center, Bethesda, Maryland, United States of America; 24 Duke Clinical Research Institute, Durham, North Carolina, United States of America; Washington Hospital Center, UNITED STATES

## Abstract

Although metabolic syndrome is associated with increased risk of cardiovascular disease and events, its added prognostic value beyond its components remains unknown. This study compared the prevalence, severity of coronary artery disease (CAD), and prognosis of patients with metabolic syndrome to those with individual metabolic syndrome components. The study cohort consisted of 27125 consecutive individuals who underwent ≥64-detector row coronary CT angiography (CCTA) at 12 centers from 2003 to 2009. Metabolic syndrome was defined as per NCEP/ATP III criteria. Metabolic syndrome patients (n=690) were matched 1:1:1 to those with 1 component (n=690) and 2 components (n=690) of metabolic syndrome for age, sex, smoking status, and family history of premature CAD using propensity scoring. Major adverse cardiac events (MACE) were defined by a composite of myocardial infarction (MI), acute coronary syndrome, mortality and late target vessel revascularization. Patients with 1 component of metabolic syndrome manifested lower rates of obstructive 1-, 2-, and 3-vessel/left main disease compared to metabolic syndrome patients (9.4% vs 13.8%, 2.6% vs 4.5%, and 1.0% vs 2.3%, respectively; p<0.05), while those with 2 components did not (10.5% vs 13.8%, 2.8% vs 4.5% and 1.3% vs 2.3%, respectively; p>0.05). At 2.5 years, metabolic syndrome patients experienced a higher rate of MACE compared to patients with 1 component (4.4% vs 1.6%; p=0.002), while no difference observed compared to individuals with 2 components (4.4% vs 3.2% p=0.25) of metabolic syndrome. In conclusion, Metabolic syndrome patients have significantly greater prevalence, severity, and prognosis of CAD compared to patients with 1 but not 2 components of metabolic syndrome.

## Introduction

By the National Cholesterol Education Program Adult Treatment Panel III (NCEP/ATP III), metabolic syndrome (MetS) is defined by the presence of at least 3 components of obesity, dyslipidemia, hypertension or treated hypertension, and elevated fasting plasma glucose levels [1,2]. The presence of MetS is believed to manifest through dysregulation of energy utilization established through insulin resistance. An array of studies has observed worsened cardiovascular prognosis and heightened mortality for individuals with MetS [3–7]. However, the various definitions of MetS, the presence of multiple subtypes of MetS with varying treatment strategies, as well as its pathogenic uncertainty has elicited questions as to whether the presence of MetS confers incremental risk of major adverse cardiovascular events (MACE) over the sum of its parts [7–10].

Coronary computed tomography angiography (CCTA) is a noninvasive diagnostic tool that has high diagnostic performance both for detection and exclusion of CAD [11]. Multicenter studies have demonstrated a prognostic utility for individuals with CCTA-identified CAD [12–14]. In this present study of individuals undergoing CCTA, we sought to determine whether or not MetS is predictive of CAD prevalence, extent and severity, and incident MACE beyond that of its individual components.

## Materials and Methods

The COronary CT Angiography EvaluatioN For Clinical Outcomes: An InteRnational Multicenter (CONFIRM) Registry is a dynamic, prospective, international, multicenter, observational registry of 27125 consecutive patients who underwent ≥64 detector row CCTA for suspected CAD at 12 centers from 2003 to 2009. The study rationale and design has been previously described [15].

Each center obtained approval from an ethics or institutional review board when required. Before CCTA, we prospectively collected information on presence of CAD risk factors. Hypertension was defined as history of high blood pressure or active treatment with anti-hypertensive medications. Diabetes was defined by a previous diagnosis of elevated fasting plasma glucose ≥126 mg/dl and/or use of insulin or hypoglycemic agents. Dyslipidemia was defined as known but untreated dyslipidemia or treatment with lipid lowering medications, and included elevated levels of low density lipoproteins, elevated triglycerides, or low levels high density lipoproteins. Body mass index (BMI) was utilized as a measure of obesity, and was calculated as weight in kilograms divided by square of height in meters, with value ≥30 considered obese.

CCTA was performed using multiple scanner platforms (Light speed VCT, GE Healthcare, Milwaukee, WI; Somatom Definition CT, Siemens, Ehrlangen, Germany; Somatom Definition Flash CT, Siemens, Ehrlangen, Germany). Contrast (80 to 140 ml) was injected, and whole-volume image acquisition was completed in a single breath-hold. The scan parameters were 64×0.625/0.750 mm collimation and tube voltage 100 or 120 kVp, and the tube current was assigned based on body size and scanner platform.

Helical or axial scan data were obtained with retrospective or prospective electrocardiogram gating, respectively. Acquired image data were initially reconstructed in mid-diastole (always) and end-systole (when available) and the phase with the least amount of coronary artery motion was chosen for analysis. Reconstructed data were evaluated by highly experienced readers (Level III equivalent and/or board certified in CCTA) using all necessary post processing techniques to determine the presence of CAD in any visible segment ≥ 2 mm in diameter. A 16-segment American Heart Association coronary artery tree model was used [16]. In each coronary artery segment, coronary atherosclerosis was defined as tissue structures >1 mm^2^ that existed either within the coronary artery lumen or adjacent to the coronary artery lumen that could be discriminated from surrounding pericardial tissue, epicardial fat, or the vessel lumen itself and identified in ≥2 planes. Coronary lesions were quantified for luminal diameter stenosis by visual estimation and were graded as none (0% luminal stenosis), non-obstructive (1–49%) and obstructive (≥50%). Plaque composition in each coronary segment was reported as calcified, non-calcified, or partially calcified [17].

Plaque severity was graded at per-vessel and per-patient level. For purposes of classification for per-vessel analyses, we considered four arterial territories: (1) left main (LM) artery, (2) left anterior descending (LAD) artery, (3) left circumflex (LCx) artery, and (4) right coronary artery (RCA). Obstructive CAD in the diagonal branches, obtuse marginal branches, and posterolateral branches was considered to be part of the LAD, LCx, and RCA system, respectively. The posterior descending artery (PDA) was considered as part of the RCA or LCx system, depending upon coronary artery dominance.

MetS was defined as the presence of 3 or more of the following: (1) diabetes mellitus or use of hypoglycemics, (2) dyslipidemia, (3) hypertension or use of anti-hypertensive medications, or (4) elevated BMI≥30. We used a history of diabetes or use of insulin/oral hypoglycemic medication as a marker for impaired fasting blood glucose, BMI ≥30 as a marker for waist circumference, and hypertension or use of anti-hypertensive medications as a marker for elevated blood pressure [18,19].

MACE were defined by a component endpoint of all-cause mortality, non-fatal myocardial infarction (MI) or unstable angina (UA), and late target revascularization ≥90 days after CCTA in accordance with ACC/AHA guidelines and the ESC/ACCF/AHA/WHF Universal Definition of Myocardial Infarction [20–22]. Follow-up for MACE was performed at each institution by a dedicated physician and/or research nurse. Sites within the United States (US) ascertained death by query or by the National Death Index. In non-US sites, ascertainment of death was determined by direct interview and/or telephone contact, and/or review of medical records. Additional event ascertainment including MI, was performed at certain sites by direct interview, telephone contact, or review of medical records.

Continuous variables are presented as mean ± SD and were evaluated using a Student unpaired t*-*test or a Wilcoxon rank-sum test, as appropriate. Categorical variables are presented as frequencies with percentages and evaluated using the Pearson Chi-square test or Fisher Exact test where there were cell counts <6. Comparisons were made between those MetS patients versus patients without MetS. Statistical significance was accepted for two-sided p-values <0.05. All calculations were performed using STATA version 11 (StataCorp, College Station, Texas). For outcome statistics, annualized event rates were calculated by dividing the number of MACE by person years, and with MACE compared using the log-rank test and visualized using Kaplan-Meier graphs. Cox regression was used to evaluate MetS subgroups, adjusting for age, gender, and chest pain type. The assumption of proportion hazards was verified using Schoenfeld residuals.

Propensity scores were derived using logistic regression to match the MetS patients to patients without MetS, both for those with 1 component of MetS as well as for those with 2 components of MetS. Propensity scores accounted for age, gender, smoking, and family history; and consisted of the resulting predicted probabilities of a logistic regression model predicting the presence of MetS versus those with individual components of MetS. The resulting propensity scores were then applied 1:1 to match every patient without MetS to a corresponding patient with MetS using the Mahalanobis nearest-neighbor matching algorithm [23]. This matching resulted in 1:1:1 matching of 690:690:690 patients with 1 or 2 components of MetS and MetS, respectively.

## Results

From 27125 consecutive patients undergoing CCTA, 3900 patients with any clinical component of MetS were identified, with 690 patients fulfilling the diagnostic criteria for MetS (Table 1). Propensity score matching resulted in mean caliper differences of 0.0004±0.001 and 0.0005±0.001 for patients with 1 or 2 components of MetS, respectively. Standardized differences were <0.1. Among matched patients with ≥1 component of MetS and MetS, no differences were observed for age, sex, family history of CAD, smoking status, dyslipidemia, and presenting symptom type; although hypertension, diabetes mellitus and obesity were more common for patients with MetS (p<0.001) (Table 1).

**Table 1:** Baseline characteristics of matched individuals with MetS versus those with individual components of MetS.

Variable	MetS^a^	No MetS, 1 component	p-Value	No MetS, 2 components	p-Value
**N**	690	690		690	
Age (years)*	57.6±11.2	57.3±11.6	0.58	58.0±11.5	0.57
Male Sex*	52.2%	51.7%	0.87	51.2%	0.71
Family hx of CAD*	27.8%	28.8%	0.68	27.8%	1.00
Smoker*	16.5%	14.1%	0.20	15.7%	0.66
**Hyperlipidemia**	53.0%	50.7%	0.40	51.4%	0.55
**Hypertension**	70.4%	47.2%	<0.001	58.0%	<0.001
**Diabetes Mellitus**	33.9%	2.3%	<0.001	13.5%	<0.001
**BMI** ^**b**^ (**kg/m** ^**2**^)	30.3±6.1	25.2±3.8	<0.001	27.6±5.0	<0.001
**Chest Pain Symptoms**					
**Asymptomatic**	39.2%	38.9%	0.92	38.5%	0.80
**Non Cardiac**	4.7%	3.5%	0.29	3.7%	0.36
**Atypical Angina**	41.8%	45.0%	0.24	45.5%	0.18
**Typical Angina**	14.3%	12.5%	0.34	12.3%	0.29

^a^ MetS = metabolic syndrome

^b^ BMI = body mass index

*Matched variables

By CCTA, extent and severity of CAD was significantly different for patients with versus without MetS. For individuals with 1 component of MetS, patients with MetS manifested a lower rate of normal coronaries, and higher rates of non-obstructive and obstructive CAD; as well as those with more 1-vessel, 2-vessel, 3-vessel/left main disease and a higher stenosis score (Table 2). When compared to patients with 2 components of MetS, individuals with MetS demonstrated lower rates of normal coronary arteries and higher rates of obstructive CAD. For patients with 2 components of MetS, the prevalence of non-obstructive plaque, presence of obstructive 1-vessel, 2-vessel and 3-vessel/LM disease were not significantly different when compared to patients with MetS (p>0.05).

**Table 2:** Comparison of prevalence, extent and severity of coronary artery disease in matched individuals with versus without MetS based on number of individual components of MetS.

Variable	MetS^c^	No MetS, 1 component	p-Value	No MetS, 2 components	p-Value
**Normal (%)**	43.8%	58.3%	<0.001	53.3%	<0.001
**Non Obstructive (%)**	35.6%	30.0%	0.03	32.0%	0.16
**Obstructive (%)**	20.6%	11.7%	<0.001	14.6%	0.003
**Segment Stenosis Score**	2.5±3.9	1.5±2.6	<0.001	1.9±3.1	<0.001
**Obstructive 1 VD (%)**	13.8%	8.4%	0.001	10.5%	0.06
**Obstructive 2 VD (%)**	4.5%	2.5%	0.04	2.8%	0.08
**Obstructive 3 VD** ^**a**^ **/LM** ^**b**^ **(%)**	2.3%	0.7%	0.02	1.3%	0.16

^a^ VD = vessel disease

^b^ LM = left main

^c^ MetS = metabolic syndrome

At median follow-up of 2.5 years (interquartile range 1.5–3.5 years), 63 MACE events occurred. The presence of MetS was associated with higher rates and risk of mortality and MACE but similar rates and risk of MI (Table 3, Table 4).

**Table 3:** Incident MACE rates for matched patients with versus without MetS.

Annualized Event Rates	All-Cause Mortality	Myocardial Infarction	MACE^b^
**No MetS** ^**a**^ **(n = 1380)**	0.5%	0.2%	0.8%
**MetS (n = 690)**	1.2%	0.1%	1.9%
**p-Value**	0.007	0.80	0.002
**No MetS, 1 component (n = 690)**	0.3%	0.1%	0.6%
**No MetS, 2 components (n = 690)**	0.7%	0.3%	1.1%
**MetS (n = 690)**	1.2%	0.1%	1.9%
**p-Value (for trend)**	0.009	0.22	0.003

^a^ MetS = metabolic syndrome

^b^ MACE = major adverse cardiac events

**Table 4:** Hazards ratios for incident MACE for patients with versus without MetS.

Hazard Ratios	Death (95% CI)	p-Value	MI^b^ (95% CI^c^)	p-Value	MACE (95% CI)	p-Value
**No MetS** ^**a**^ **(n = 1380)**	1.0	Reference	1.0	Reference	1.0	Reference
**MetS (n = 690)**	2.4 (1.2–4.7)	0.009	0.8 (0.2–4.0)	0.80	2.2 (1.3–3.7)	0.003
**No MetS, 1 components (n = 690)**	1.0	Reference	1.0	Reference	1.0	Reference
**No MetS, 2 components, (n = 690)**	2.5 (0.9–7.0)	0.09	5.2 (0.6–44.5)	0.13	2.0 (0.9–4.2)	0.08
**MetS (n = 690)**	4.2 (1.5–11.2)	0.005	2.5 (0.2–27.4)	0.46	3.3 (1.6–6.7)	0.001

^a^ MetS = metabolic syndrome

^b^MI = myocardial infarction

^c^CI = confidence interval

## Discussion

This study from the CONFIRM registry is the first prospective, multicenter analysis to provide data on the per-patient, per-vessel, and per-segment extent, prevalence and severity of CAD by CCTA in individuals with versus without MetS who were similar in age, sex, family history of premature CAD, and smoking history. We observed a higher prevalence, extent and severity of CAD and MACE rates in patients with versus without MetS, a finding that was associated with worsened prognosis for MACE and their individual components. When further stratified, however, the higher prevalence of CAD and worsened prognosis for MetS patients versus those without MetS was limited to patients with a single clinical component of MetS and was no different for those individuals with 2 clinical components of MetS.

The coexistence of clinical CAD risk factors that include impaired fasting glucose, obesity, and atherogenic dyslipidemia has been termed MetS, a condition that is defined as an “aggregate of symptoms and signs associated with any morbid process, and constituting together the picture of the disease” [24]. Within the context of MetS, an underlying unifying pathology of insulin resistance and resultant energy dysregulation has been demonstrated to be associated with increased risk for MACE and death [3–7]. The degree to which MetS confers this risk is variable, with prior investigations suggesting that the components of MetS, when present together, results in a risk that is greater than the sum of its parts [25–28].

Yet little data exists when comparing the prevalence, extent and severity of CAD, as well as risk of downstream adverse clinical events for patients with MetS versus those with underlying individual components of MetS, particularly in individuals who are similar in other traditional CAD risk factors. To our knowledge, these data represent the first to directly compare these findings in patients with versus without MetS in a population similar in age, sex, smoking, and family history of premature CAD. When examined against patients with MetS, we observed a differential CAD profile and risk of downstream clinical adverse events for patients with 1 but not 2 components of MetS.

These present data add to a body of literature that has observed inconsistent observations regarding the concept of MetS as a clinical syndrome confers risk beyond its individual clinical components. As an example, in the Atherosclerosis Risk in Communities (ARIC) study, metabolic syndrome was observed to be associated with risk of 11-year cardiovascular outcomes, a finding that was nevertheless mitigated by traditional risk factor scoring using the Framingham Risk Score [29]. In contrast, patients with the Scandinavian Simvastatin Survival Study and the Air Force/Texas Coronary Atherosclerosis Prevention Study demonstrated increase risk of major coronary events above and beyond Framingham Risk Scores [30]. Our study differs and is directly additive to these prior investigations in that we examined risk for patients undergoing non-invasive angiography, and on a backdrop of CAD risk factor similarity.

This study is not without limitations. First, the study includes patients undergoing clinical indicated CCTA studies and whether the present results can be extrapolated to a population-based cohorts remains unknown. Furthermore, despite the prospective multinational nature of study in this patient cohort, it nevertheless remains observational in nature and the potential biases associated with all such studies cannot be discounted. Third, we employed BMI, diabetes and hypertension as surrogates for components of MetS and whether greater precision would have been observed had measures of waist circumference, triglycerides, low-density lipoproteins, high-density lipoproteins and fasting plasma glucose been available. Fourth, the downstream effects of the presence of MetS and its individual components are unknown, including the use of medical therapies, lifestyle modification, and interventional procedures. Finally, while our study represents the largest one of its kind to date, the number of MACE observed were generally low and therefore our study might be underpowered to show a significant difference between those who presented with 2 MetS risk factors and patients with MetS.

## Conclusion

Prevalence, extent, severity of CAD, and risk of MACE rates are significantly increased among patients with MetS compared to those with only 1 component of MetS. This finding is observed for patients with a single clinical component of MetS and not for patients with two components of MetS.
